# Novel Functional Role of NK3R Expression in the Potentiating Effects on Somatolactin α Autoregulation in grass carp pituitary cells

**DOI:** 10.1038/srep36102

**Published:** 2016-10-27

**Authors:** Guangfu Hu, Mulan He, Anderson On Lam Wong

**Affiliations:** 1 College of Fisheries, Key Laboratory of Freshwater Animal Breeding, Freshwater Aquaculture Collaborative Innovation Center of Hubei Province, Huazhong Agricultural University, Wuhan, 430070, China; 2 School of Biological Sciences, University of Hong Kong, Hong Kong, China

## Abstract

In our previous study, NKB/NK3R system has been shown to act at the pituitary level to up-regulate SLα synthesis and secretion in grass carp. However, whether NK3R expression can serve as a regulatory target at the pituitary level and contribute to NKB interactions with other SLα regulators is still unclear. In current study, using grass carp pituitary cells as a model, we have a novel finding that co-treatment of SLα/SLβ with carp TAC3 gene products, could induce a noticeable enhancement in SLα mRNA expression and these potentiating effects occurred with a parallel rise in NK3R transcript level after SLα/SLβ treatment. Interestingly, the stimulatory effects of SLα/SLβ on NK3R gene expression could be further potentiated by co-treatment with IGF-I/-II and simultaneous exposure of carp pituitary cells to SLα/SLβ and IGF-I/-II in the presence of TAC3 gene products was found to markedly elevated SLα mRNA expression (20 fold increase) and this synergistic stimulation was mediated by cAMP/PKA-, PLC/PKC- and Ca^2+^ -dependent cascades functionally coupled with NK3R activation. These findings suggest that local release of SLα via functional interactions with IGF-I/-II and TAC3/NK3R system may constitute a potent stimulatory signal for SLα gene expression in the carp pituitary via up-regulation of NK3R expression.

Somatolactin (SL), the latest member of growth hormone (GH)/prolactin (PRL) family, is a fish-specific hormone released from the neurointermediate lobe (NIL) of the posterior pituitary^1^. Two isoforms of SL, SLα and SLβ, have been identified in fish pituitary, e.g., in zebrafish^2^, salmon^3^, and grass carp^4^, and suspected to have overlapping and yet distinct functions^5^. To date, SL has been shown to be involved in diverse functions in fish models, including chromatophore proliferation and differentiation^6^, pigment aggregation^7^, inflation of swim bladder during embryo development^5^, reproduction^3^, stress responses^8^, lipid metabolism^9^, and osmoregulation^10^. At the pituitary level, somatolactin secretion and gene expression are known to be under the control by various hypothalamic factors, e.g., dopamine^11^, GnRH^12^,^13^, PACAP^4^, neurokinin B (NKB)^14^, MCH^15^ and octadecdaneuropeptide^16^. Recently, insulin-like growth factors (IGFs) have also been identified as potent stimulators for SLα and SLβ secretion and gene expression in grass carp pituitary cells^17^. At the pituitary level, “somatolactin autoregulation” via autocrine/ paracrine effects of SLα and SLβ released locally within the pituitary has also been reported in grass carp^7^. However, the functional interactions of local release of SL with other SL regulators have not been examined and still awaited for further investigation.

NKB is the gene product of tachykinin 3 (TAC3), which is a member of the tachykinin family with pleiotropic functions, including the control of smooth muscle contraction in the gastrointestinal tract^18^, fluid secretion in the gut epithelium^19^, vasodilating effect for blood pressure modulation^20^ and stimulating effect on sperm movement^21^. The biological actions of NKB are mediated mainly by the Type 3 neurokinin receptor (NKR), namely NK3R, which is a member of the rhodopsin-type class I group G protein coupled receptor^22^ and functionally coupled with cAMP/PKA-23, PLC/IP_3_/PKC-24, and Ca^2+^ -dependent^25^ signaling pathways. In recent years, the functional role of NKB in puberty onset^26^ and human fertility has aroused a lot of interest in the field of reproductive biology, mainly due to the findings that loss-of-function mutations in NKB or its receptor NK3R can lead to hypogonadotropic hypogonadismor and even infertility in human subjects^27^,^28^,^29^. Based on the studies in mammals (e.g., rodent and sheep), NKB was found to be a key regulator for GnRH pulsatility and downstream LH release via NK3R activation in kisspeptin neurons located in the arcuate nuclei within the hypothalamus^30^,^31^. Similar investigations have been recently extended to fish models with the novel findings that the TAC3 gene in fish species, e.g., in zebrafish^32^,^33^,^34^, goldfish^35^, tilapia^36^ and grass carp^14^, not only encodes NKB as the gene product, but also the mature peptide of a new member of tachykinin called NKB-related peptide (NKBRP). Similar to NKB, NKBRP was also effective in stimulating LH release, e.g., in zebrafish^32^, goldfish^35^ and tilapia^36^, suggesting the reproductive function of TAC3 gene products was well conserved throughout vertebrate evolution. Based on our recent *in vitro* studies in grass carp pituitary cells, interestingly, NKB and NKBRP were found to have no effect on LH secretion and LHβ & GtHα gene expression at the pituitary level but rather serve as novel stimulators for prolactin (PRL) and SLα secretion and gene expression via differential activation of NK2R and NK3R expressed in the carp pituitary^14^. The investigation on SLα regulation by TAC3 gene products in the carp model have become more exciting with the recent demonstration that IGF-I/-II could act in a synergistic manner with TAC3 gene products, namely NKB and NKBRP, to up-regulate SLα gene expression at the pituitary level and this potentiating effect could be paralleled with the concurrent rise in NK3R expression induced by IGF-I/-II treatment. These new findings demonstrated for the first time that NK3R expression at the pituitary level could serve as a regulatory target for modulation of pituitary hormone gene expression in vertebrate species.

In this study, using primary culture of grass carp pituitary cell as a model, the functional interactions between somatolactin autoregulation with IGF-I/-II and TAC3 gene products on SLα gene expression were examined in the carp pituitary with focus on the role of NK3R expression as a regulatory target at the pituitary level. As a first step, co-treatment of SLα/SLβ with either IGF-I/-II alone or TAC3 gene products alone including NKBa and NKBRPa (the gene products of carp TAC3a gene) or with a combination of both were performed to examine their effects on SLα mRNA expression in carp pituitary cells. The potentiating effects observed with SL/IGFs and SL/TAC3 gene product co-treatment on SLα gene expression were correlated with parallel changes of NK3R mRNA expression induced by SLα/SLβ treatment alone or in combination with IGF-I/-II co-treatment. Using a pharmacological approach, the signal transduction mechanisms involved in SLα and SLβ induction of NK3R mRNA expression were elucidated and the functional role of NK3R expression and the post-receptor signaling pathways coupled with NK3R activation in the potentiating effects on SLα mRNA expression observed with cotreatment of IGF-I/-II and TAC3 gene products was also confirmed at the pituitary cell level. Our studies for the first time provide evidence that local release of SLα and SLβ could interact with IGF-I/-II and TAC3 gene products to up-regulate SLα gene expression in the carp pituitary via stimulation of NK3R expression at the pituitary level.

## Results

### Synergistic effects of somatolactin and TAC3a gene products on SLα mRNA expression

**Figure 1:**
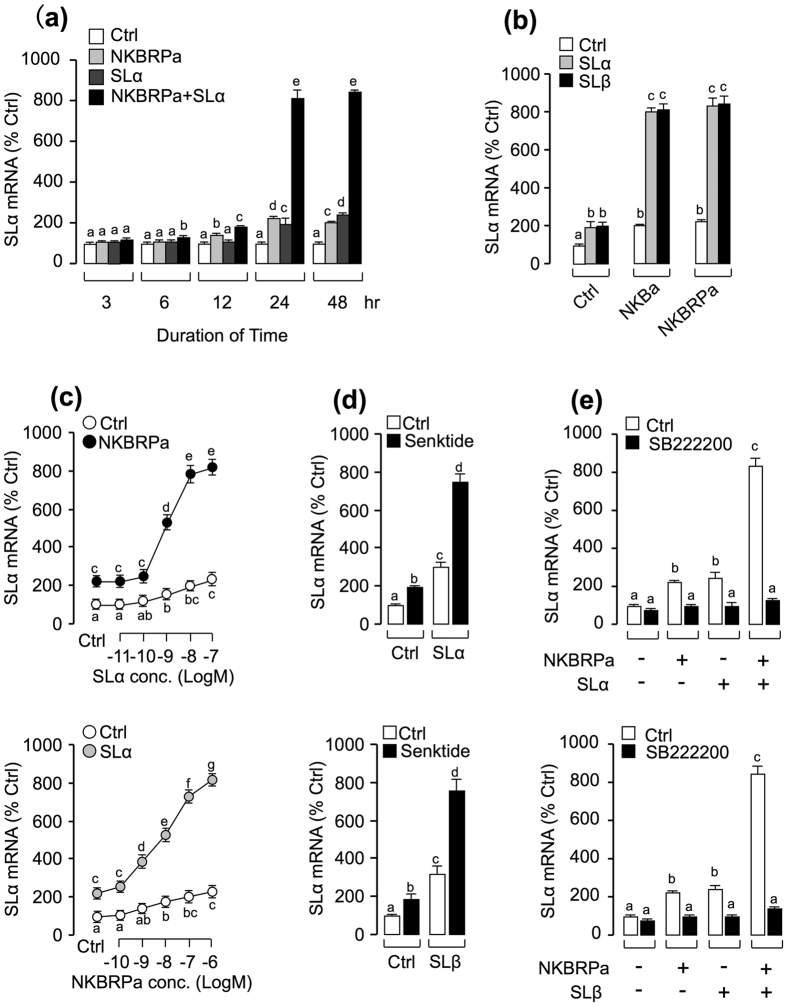
Synergism between NKBRPa and SLα/β in stimulation of SLα gene expression. **(a)** Time course of NKBRPa (1 μM), SLα (30 nM), and NKBRPa (1 μM)^+^ SLα (30 nM) on SLα mRNA expression in carp pituitary cells. **(b)** Synergistic effect of SLα/β with NKBa and NKBRPa in stimulation of SLα mRNA expression. In this experiment, carp pituitary cells were incubated for 24-hr with SLα/β (30 nM) co-treatment with NKBa, NKBRPa, and senktide (1 μM). **(c)** Effect of SLα concentration (0.01–100 nM) on basal and NKBRPa (1 μM)-induced SLα mRNA expression in carp pituitary cells. **(d)** Effects of NKBRPa concentration (0.1–1000 nM) on basal and SLα(30 nM)-induced SLα transcript levels in carp pituitary cells. **(e)** Synergistic effect of senktide (1 μM) with SLα and SLβ in stimulation of SLα mRNA expression. Receptor specificity for SLα regulation by SLα/β with NKBRPa. In this experiment, carp pituitary cells were challenged for 24-hr with SLα (30 nM)^+^ NKBRPa (1 μM) or SLβ (30 nM)^+^ NKBRPa (1 μM) in the presence or absence of NK3R antagonist SB222200 (10 μM). After drug treatment, total RNA was isolated for real-time PCR of SLα mRNA. In the data present (mean ± SEM), the groups denoted by different letters represent a significant difference at p < 0.05 (ANOVA followed by Dunnett’s test).

Given that (i) two somatolactin isoforms, SLα and SLβ, have been previously shown to trigger SLα secretion and gene expression at the pituitary level^7^, and (ii) TAC3a gene products could also stimulate SLα secretion and gene expression via activation of NK3R in carp pituitary cells^14^, we examined the functional interaction between TAC3a gene products and SLs in their stimulatory activity on SLα gene expression. As shown in Fig. 1a, static incubation with SLα (30 nM) and NKBRPa (1 μM) alone were both effective in elevating SLα mRNA expression in carp pituitary cells in a time-dependent fashion. Interestingly enough, the stimulatory effect on SLα mRNA expression was markedly enhanced (up to 8 fold basal) especially after 24–48 hr of drug treatment with simultaneous exposure to both SLα (30 nM) and NKBRPa (1 μM). In the single-dose experiment with drug treatment fixed at 24 hr, the potentiating effect (up to 8 fold basal) could still be observed with co-treatment of either SLα or SLβ (30 nM) with the carp TAC3a gene products including NKBa (1 μM) and NKBRPa (1 μM), respectively (Fig. 1b). In the parallel experiments, the synergistic action between NKBRPa and SLα was also confirmed by a concentration-response study. As shown in Fig. 1c, NKBRPa (1 μM)-induced SLα mRNA expression was found to be aggravated in a dose-dependent manner with cotreatment of increasing level of SLα (0.01–100 nM). Similar dose-dependence of the potentiating effect was also noted in the reciprocal experiment with cotreatment of SLα (30 nM) with increasing levels of NKBRPa (0.1–1000 nM). In the case of SLβ regulation, IGF-I, SLα and SLβ alone could all trigger SLβ mRNA expression in grass carp pituitary cells, but static incubation with NKBa or NKBRPa were both not effective in elevating SLβ mRNA expression and secretion significantly. In addition, cotreatment with NKBa/NKBRPa and SLα could not potentially increase SLβ transcript levels (Fig.S1). To establish the functional link between SLα potentiation and NK3R at the pituitary level, the NK3R agonist senktide (1 μM) was substituted for NKBa and NKBRPb in the potentiating study with SLα co-treatment. As shown in Fig. 1d, NK3R activation with senktide was found to mimic the synergistic effects of TAC3a gene products on SLα mRNA expression when give together with either SLα (30 nM) or SLβ (30 nM). Besides, co-treatment with NK3R antagonist SB222200 (10 μM) not only could reduce the stimulatory actions on SLα mRNA expression induced by SLα (30 nM)/SLβ (30 nM) and NKBRPa (1 μM) alone, but also significantly suppressed the potentiating effect induced by SLα/SLβ and NKBRPa co-treatment (Fig. 1e). These results indicated that the synergistic effect of SL and TAC3 gene products on SLα mRNA expression is dependent on NK3R activation.

### Up-regulation of NK3R gene expression by SLα/β in grass carp pituitary cells

**Figure 2:**
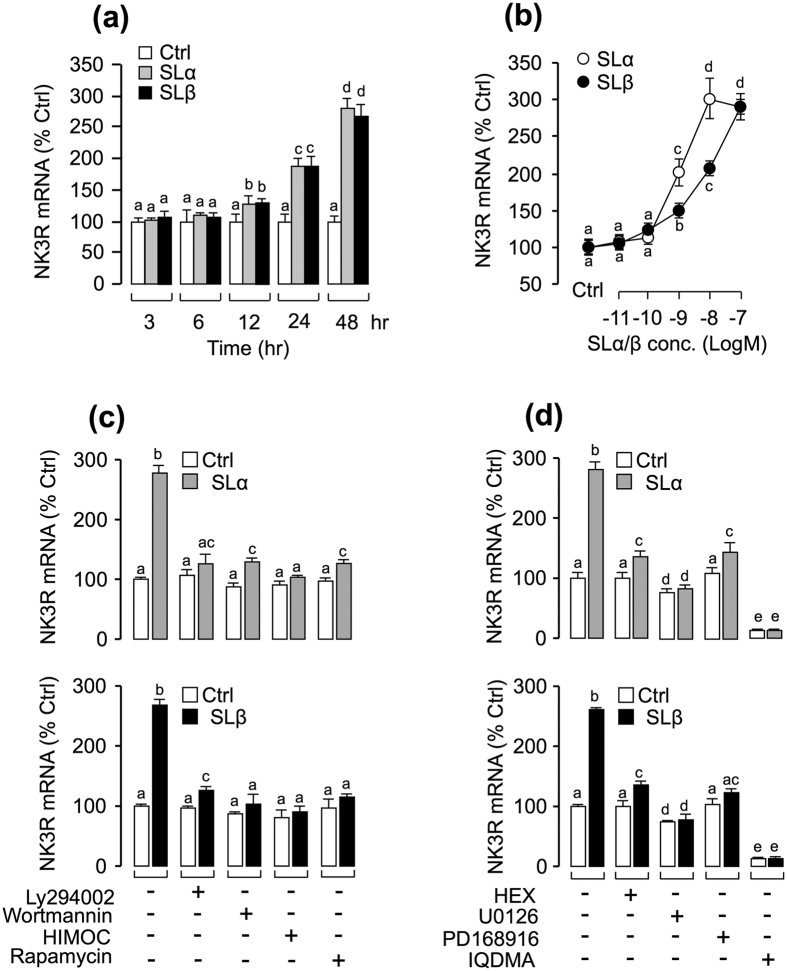
Up-regulation of NK3R gene expression by SLα/β in grass carp pituitary cells. **(a)** Time course of carp SLα (30 nM) and SLβ (30 nM) treatment on NK3R mRNA expression in carp pituitary cells. **(b)** 24-hr incubation with increasing levels of SLα or SLβ (0.01–100 nM) treatment on NK3R mRNA expression in carp pituitary cells. **(c)** Effects of 24-hr co-treatment with the PI_3_K inhibitor Ly294002(10 μM) and wortmannin (1 μM), Akt inhibitor HIMOC (10 μM) and mTOR inhibitor rapamycin (20 nM) on SLα (30 nM)- or SLβ (30 nM)-induced NK3R transcript expression in carp pituitary cells. **(d)** Effects of 24-hr co-treatment with the JAK_2_ inhibitor HEX (50 μM), STAT_5_ inhibitor IQDMA (50 μM), MEK_1/2_ inhibitor U0126 (10 μM) and p38 MAPK inhibitor PD169816 (10 μM) on SLα (30 nM)- or SLβ (30 nM)-induced NK3R mRNA expression. After drug treatment, total RNA was isolated for real-time PCR of NK3R mRNA. In the data present (Mean ± SEM), the groups denoted by different letters represent a significant difference at p < 0.05 (ANOVA followed by Dunnett’s test).

According to our previous study, IGFs could strongly enhance NKB-induced SLα mRNA expression through the up-regulation of NK3R expression in carp pituitary cells. To test the hypothesis that NK3R modulation may have occurred during SL potentiation of NKB-induced SLα expression, primary culture of grass carp pituitary cells were challenged with recombinant carp SLα and SLβ, respectively. Our time-course experiment revealed that SLα/SLβ (30 nM) could both significantly trigger NK3R mRNA expression from 12 hr to 48 hr in a time-dependent fashion (Fig. 2a). In parallel dose-dependent studies, a 24-hr incubation with increasing levels of SLα or SLβ (0.1–100 nM) also elevated NK3R mRNA expression in a dose-dependent manner (Fig. 2b). To further elucidate the signal transduction mechanisms for NK3R regulation by SLs, various pharmacological blockers targeting different pathways were recruited. As shown in Fig. 2c, SLα- or SLβ-induced NK3R transcript expression could be abolished by simultaneous incubation with the PI_3_K inhibitor wortmannin (1 μM), Akt inhibitor HIMOC (10 μM), or mTOR inhibitor rapamycin (20 nM). Similar results were also observed by preventing PI_3_K activation using another PI_3_K inhibitor Ly294002 (10 μM; Fig. 2c). Given that SL activation of MAPK cascades has been reported in grass carp pituitary cells^7^, the functional role of MAPK cascades on SL-induced NK3R gene expression were also tested in carp pituitaries. As shown in Fig. 2d, SLα- or SLβ -induced NK3R mRNA expression were found to be sensitive to the blockade by the MEK_1/2_ inhibitor U-0126 (10 μM) or p_38_MAPK inhibitor PD-169816 (10 μM). In the parallel experiments, SLα- or SLβ -induced NK3R mRNA expression could be attenuated or totally abolished by simultaneous treatment with the JAK_2_ inhibitor HEX (50 μM; Fig. 2d), and STAT_5_ inhibitor IQDMA (50 μM; Fig. 2d), respectively.

### Synergistic effects of IGF and SL on NK3R and SLα gene expression

**Figure 3:**
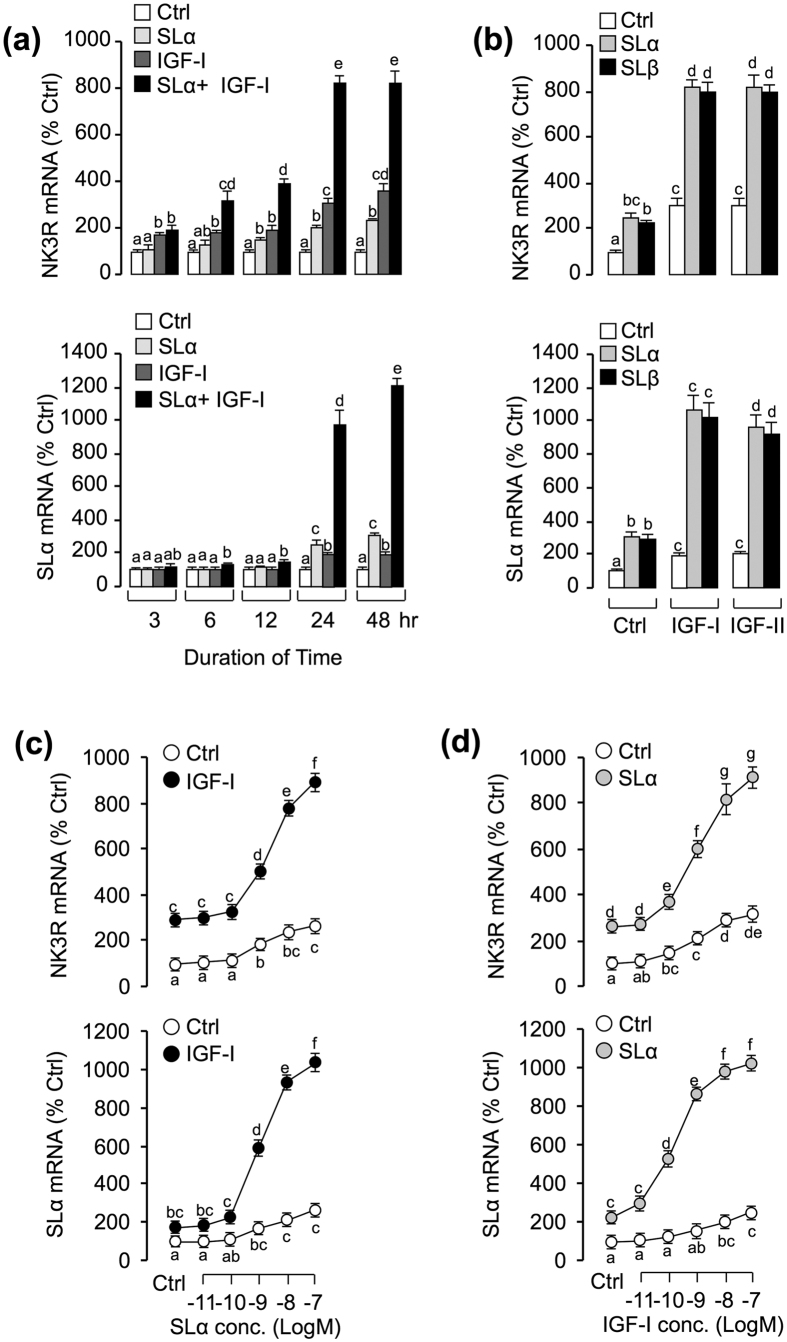
Functional interaction between SL and IGF on the regulation of NK3R and SLα gene expression. **(a)** Time course of carp SLα, IGF-I and SLα^+^ IGF-I on NK3R and SLα mRNA expression. In this experiment, carp pituitary cells were incubated from 3 to 48 hr with SLα (30 nM) alone, IGF-I (50 nM) alone and SLα (30 nM)^+^ IGF-I (50 nM). **(b)** Synergistic effects of SLα/β and IGF-I/-II on the simulation of NK3R and SLα mRNA expression. In this case, carp pituitary cells were challenged for 24-hr with SLαor SLβ (30 nM) co-treatment with either IGF-I or IGF-II (50 nM). **(c)** Effect of SLα (0.01–100 nM) treatment on basal and IGF-I (50 nM)-induced NK3R and SLα mRNA expression in carp pituitary cells. **(d)** Effects of IGF-I (0.01–100 nM) treatment on basal and SLα(30 nM)-induced NK3R and SLα transcript levels in carp pituitary cells. After drug treatment, total RNA was isolated for real-time PCR of NK3R and SLα mRNA. In the data present (mean ± SEM), the groups denoted by different letters represent a significant difference at p < 0.05 (ANOVA followed by Dunnett’s test).

Given our recent studies that IGFs and SLs were both found to be effective in stimulating SLα and NK3R gene expression in carp pituitary cells^7^,^17^, the functional interaction between SLs and IGFs in the regulation of SLα and NK3R gene expression were examined in carp pituitary cells. As shown in Fig. 3a, SLα (30 nM) and IGF-I (50 nM) treatment alone could both significantly elevate NK3R and SLα mRNA expression in carp pituitary cells in a time-dependent manner. Interestingly, the stimulatory effects on NK3R and SLα mRNA expression were significantly enhanced (up to 8 fold for NK3R and 10 fold for SLα) especially after 24–48 hr of drug treatment with co-treatment of SLα (30 nM) with IGF-I (50 nM). Following the time-course experiment, a single-dose experiment was performed at 24 hr. As shown in Fig. 3b, the potentiating effect could still be observed with co-treatment of either SLα (30 nM) or SLβ (30 nM) with IGF-I (50 nM) or IGF-II (50 nM), respectively. Besides, the functional interaction between SLα and IGF-I was further confirmed by a dose-response reciprocal reverse experiment. In this case, IGF-I-induced NK3R and SLα mRNA expression were found to be enhanced in a dose-dependent manner with simultaneous treatment with increasing concentrations of SLα (0.01–100 nM; Fig. 3c). The maximal responses occurred at 100 nM SLα (9 fold basal for NK3R and 11 fold basal for SLα). Similar concentration-dependence of the potentiating effect was also observed in the reciprocal experiment with co-treatment of SLα (30 nM) with increasing levels of IGF-I (0.01–100 nM; Fig. 3d).

### Synergistic effects of SLα, IGF-I and NKBRPa on SLα mRNA expression

**Figure 4:**
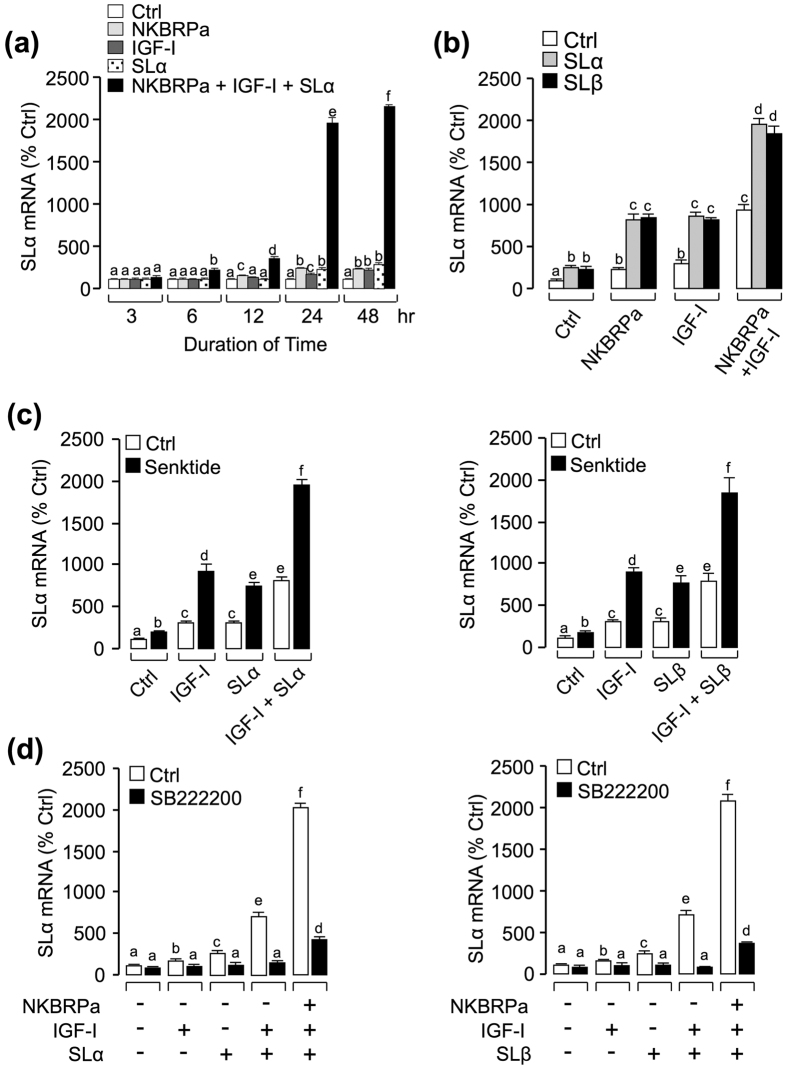
Functional interactions of SLα/β, IGF-I and NKBRPa/senktide on the regulation of SLα mRNA expression. **(a)** Time course of SLα (30 nM), NKBRPa (1 μM), IGF-I (50 nM) and SLα (30 nM)^+^ NKBRPa (1 μM)^+^ IGF-I (50 nM) treatment on SLα mRNA expression. **(b)** Synergistic effects of SLα/β with NKBRPa, IGF-I or NKBRPa^+^ IGF-I. In this case, carp pituitary cells were incubated for 24-hr with SLα or SLβ (30 nM) co-treatment with NKBRPa (1 μM), IGF-I (50 nM) or NKBRPa (1 μM)^+^ IGF-I (50 nM), respectively. **(c)** Synergistic effects of senktide with SLα/β, IGF-I or SLα/β^+^ IGF-I. In this case, carp pituitary cells were incubated for 24-hr with senktide (1 μM) co-treatment with SLα/β (30 nM), IGF-I (50 nM) or SLα/β(30 nM)^+^ IGF-I (50 nM), respectively. **(d)** Receptor specificity for SLα regulation by SLα or SLβ co-treatment with IGF-I or NKBRPa^+^ IGF-I. In this experiment, carp pituitary cells were incubated for 24-hr with SLα (30 nM)^+^ IGF-I (50 nM) and SLα (30 nM)^+^ NKBRPa (1 μM)^+^ IGF-I (50 nM) or SLβ (30 nM)^+^ IGF-I (50 nM) and SLβ (30 nM)^+^ NKBRPa (1 μM)^+^ IGF-I (50 nM) in the presence or absence of NK3R antagonist SB222200 (10 μM). After drug treatment, total RNA was isolated for real-time PCR of SLα mRNA. In the data present (Mean ± SEM), the groups denoted by different letters represent a significant difference at p < 0.05 (ANOVA followed by Dunnett’s test).

In the current study, the synergism between SL and IGF in stimulation of NK3R mRNA expression was noted in carp pituitary cells. So now we have an interesting idea, what will happen when we use a cocktail containing SL, IGF and NKB to challenge carp pituitary cells ? To answer this question, the cocktail of SLα, IGF-I and NKBRPa was used to test grass carp pituitary cells. As shown in Fig. 4a, IGF-I (50 nM), NKBRPa (1 μM) and SLα (30 nM) treatment alone could stimulate SLα mRNA up to 24 hr, however, co-treatment of the three drugs together could significantly elevate SLα mRNA from 6 hr (up to 2 fold basal) to 48 hr (up to 20 fold basal). In the single-dose experiment with drug treatment fixed at 24 hr, the potentiating effect could still be observed with co-treatment of either SLα (30 nM) or SLβ (30 nM) with NKBRPa (1 μM) and IGF-I (50 nM) (Fig. 4b). To clarify the mechanism responsible for the regulation of SLα mRNA expression by SLα/β co-treated with IGF-I and NKBRPa, a pharmacological approach was performed in carp pituitary cells. As a first step, NK3R agonist senktide (1 μM) was recruited to replace the NKBRPa in the potentiating study with IGF-I (50 nM) and SLα (30 nM). As shown in Fig. 4c, senktide could mimic the synergistic effects of TAC3 gene products on SLα regulation when simultaneous incubation with either SLα/SLβ (30 nM) or SLα (30 nM)^+^ IGF-I (50 nM)/SLβ (30 nM)^+^ IGF-I (50 nM). Besides, co-treatment with NK3R antagonist SB222200 (10 μM) not only could reduce the stimulatory actions on SLα mRNA expression induced by IGF-I (50 nM), NKBRPa (1 μM) and SLα/SLβ (30 nM) alone, but also markedly suppressed the potentiating effect induced by SLα/SLβ^+^ NKBRPa, SLα/SLβ^+^ IGF-I and SLα/SLβ^+^ IGF-I^+^ NKBRPa (Fig. 4d). These findings, taken together, suggested that the synergistic effect of SLα/β, IGF-I and NKBRPa on the stimulation of SLα mRNA expression might be mediated through the activation of NK3R, which is a G protein coupled receptor coupled with activation of AC/cAMP/PKA, PLC/IP_3_/PKC, and Ca^2+^ /CaM/CaMK-II cascades.

**Figure 5:**
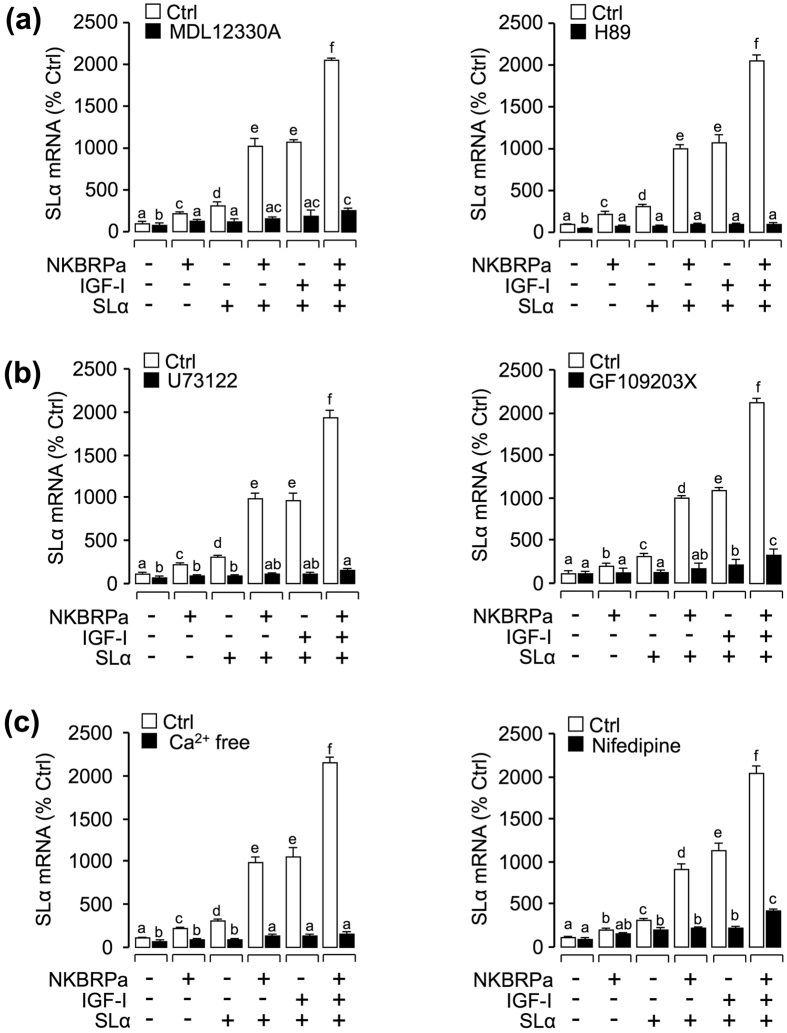
Functional role of AC/PKA, PLC/PKC and Ca^2+^ -dependent signaling pathways in the regulation of SLα mRNA expression. In this experiment, carp pituitary cells were incubated for 24 hours in the absence (control) or presence of **(a)** AC inhibitor MDL12330A (10 μM) and PKA inhibitor H89 (10 μM), **(b)** PLC inhibitor U73122 (10 μM) and PKC inhibitor (10 μM), or **(c)** Ca^2+^ free medium and VSCC blocker nifedipine (10 μM) in combination of NKBRPa (1 μM), SLα (30 nM), SLα (30 nM)^+^ NKBRPa (1 μM), SLα (30 nM)^+^ IGF-I (50 nM), or SLα (30 nM)^+^ NKBRPa (1 μM)^+^ IGF-I (50 nM). After drug treatment, total RNA was isolated for real-time PCR of SLα mRNA. In the data present (mean ± SEM), the groups denoted by different letters represent a significant difference at p < 0.05 (ANOVA followed by Dunnett’s test).

To clarify the signal transduction for the synergistic regulation of SLα mRNA expression, various pharmacological inhibitors/blockers targeting different pathways of NK3R were used. As a first step, the possible involvement of cAMP-dependent pathway was examined at the pituitary cell level. As shown in Fig. 5a, the AC-inhibitor MDL12330A (10 μM) or PKA inhibitor H89 (10 μM) could block the synergistic effects of SLα, IGF-I and NKBRPa on the induction of SLα mRNA expression. To shed light on the role of PLC-dependent cascade in the synergistic actions, SLα regulation by SLα, IGF-I and NKBRPa were tested with inhibitors for individual components of this pathway. In this case, the synergistic effects of SLα, IGF-I and NKBRPa on SLα gene expression were observed to be suppressed/abolished by simultaneous incubation with the PLC inhibitor U73122 (10 μM) or PKC inhibitor GF109203X (10 μM), respectively (Fig. 5b). To examine the possible role of Ca^2+^ -dependent cascade in SLα regulation by the cocktail containing SLα, IGF-I and NKBRPa, the synergistic effects were also tested with various inhibitors for Ca^2+^ pathway. In this case, this cocktail-induced SLα mRNA expression were found to be attenuated/abolished by incubation with Ca^2+^ free medium or co-treatment with the voltage sensitive calcium channel (VSCC) inhibitor nifedipine (10 μM), respectively (Fig. 5c).

## Discussion

At present, except for a single study in carp pituitary cells showing that SLα and SLβ play a stimulatory role in autocrine/paracrine regulation of SLα secretion and synthesis in grass carp^7^, little is known about the functional role of SLs at the pituitary level. Since (i) carp SLα and SLβ were found to be effective in triggering SLα secretion, protein production and gene expression in carp SLα cells^7^, (ii) carp NK3R was specifically expressed in SLα cells within the NIL lobe of the carp pituitary^14^, we speculate that SLs may play a role on NK3R regulation at the pituitary level, which may have a functional impact on SLα expression in carp pituitary cells. In the present study, using grass carp pituitary cells as a model, we demonstrated for the first time that SLα and SLβ can up-regulate NK3R gene expression in time- and dose-dependent manner via direct actions at the pituitary level. In fish models, SL receptor has been identified as a member of the Type I GHR family^37^, and its activation can lead to JAK_2_ recruitment and subsequent activation of STATs, MAPK, and PI_3_K pathway in grass carp pituitary cells^7^. To test the possible involvement of these signaling cascades in SL-induced NK3R, a pharmacological approach using the inhibitors for the respective pathways was used. In carp pituitary cells, the stimulatory effects on NK3R mRNA expression induced by SLα or SLβ treatment were either totally abolished or partially suppressed by the JAK_2_ inhibitor HEX, STAT_5_ blocker IQDMA, PI_3_K inhibitors wortmannin and Ly294002, Akt inhibitor HIMOC, MEK_1/2_ blocker U-0126, and P_38_ MAPK inhibitor PD169816. These results, together with our previous findings that SLα and SLβ treatment could trigger rapid phosphorylation of STAT_5_, Akt, MEK_1/2_, ERK_1/2_, MKK_3/6_, and P_38_ MAPK in carp pituitary cells^7^, suggest that SL induction of NK3R gene expression at the pituitary cell level is mediated through activation of JAK_2_/STAT_5_, PI_3_K/Akt, MEK/ERK_1/2_, and MKK_3/6_/P_38_ MAPK cascades.

In our recent *in vitro* studies, we have demonstrated that (i) SLα and SLβ could both elevate SLα mRNA expression in carp pituitary cells^7^, and (ii) carp TAC3a gene products, namely NKBa and NKBRPa, could up-regulate SLα gene expression via activation of NK3R expressed in the carp pituitary^14^. In our initial attempt of investigate the functional interactions between SLs and TAC3 gene products on SLα expression, we have the novel findings that co-treatment of SLα/SLβ with either NKBa or NKBRPa, respectively, could trigger a synergistic effect on SLα mRNA expression in a time- and dose-dependent manner. These potentiating effect could be mimicked by replacing TAC3 gene products with the NK3R agonist senktide and blocked by simultaneous incubation with the NK3R antagonist SB222200, suggesting the possible dependence of the synergistic effect on NK3R expression at the pituitary level. Together with our current finding of SLα/SLβ up-regulation of NK3R gene expression in carp pituitary cells, it raises the possibility that SL treatment may enhance the stimulatory effect of TAC3 gene products on SLα gene expression by increasing NK3R expression in the carp pituitary. In previous studies, the interactions of glucagon with GH have been reported in different species, e.g., in rat^38^ and grass carp^39^. In rat hepatocytes, cotreatment of glucagon and GH is known to have a potentiating effect on the stimulation of IGF-I mRNA expression^38^. In grass carp, our previous studies have also shown that glucagon could potentiate GH-induced IGF-I gene expression via up-regulation of GHR expression in carp hepatocytes^39^. Apparently, potentiation of the bioactivity of various members of the GH family lineage in fish species can occur by functional interactions with GPCR activation by increasing receptor expression mediated the respective stimulating influence at the cellular level.

In cancer cell models, e.g., pancreatic cancer cells, functional crosstalk of the post-receptor signaling between IGF-I/Insulin receptor with GPCR (e.g., neurotensin receptor and type I angiotensin receptor) has been reported^40^. In wound repairing of the rabbit cornea, co-treatment of IGF-I with SP is known to have a potentiating effect on the migration of corneal epithelial cells^41^,^42^. In our current study with carp pituitary cells, SLα and SLβ not only could potentiate SLα mRNA expression induced by TAC3 gene products, but also notably enhance the stimulatory effect caused by IGF-I/-II cotreatment on SLα mRNA expression. Interestingly enough, simultaneous treatment of carp pituitary cells of SLα/SLβ with IGF-I and NKBRPa was found to markedly increase basal levels of SLα mRNA up to 20 fold basal, which was much higher than the corresponding SLα responses induced by SLα/SLβ cotreatment with either IGF-I/-II (up to 8 fold basal) or TAC3 gene products NKBa and NKBRPa (up to 8 fold basal), respectively. This notable increase in the potentiating effect caused by simultaneous treatment of SLα, IGF-I and NKBRPa together also occurred with a novel finding in carp pituitary cells, in which cotreatment with SLα/SLβ could potentiate the stimulatory effect of IGFs on NK3R gene expression at the pituitary level. Similar to our results with SL cotreatment with TAC3 gene products, the highly potent synergistic effect on SLα gene expression induced by simultaneous treatment of the three stimulators together could be mimicked by substituting the NK3R agonist senktide for NKBRPa and blocked by cotreatment with the NK3R antagonist SB222200. These findings strongly suggest that the potentiating effect caused by the three SL stimulators is highly depended on NK3R expression in the carp pituitary. NK3R is a member of the rhodopsin-type class I type G-protein coupled receptors (GPCRs), and in mammals its activation can trigger intracellular signaling via G_o_ and G_q/11_^25^,^43^ followed by cAMP production^44^,^45^, PLC-dependent PI hydrolysis^23^,^24^, mobilization of IP_3_-sensitive intracellular Ca^2+^ ([Ca^2+^]_i_)^24^ and extracellular Ca^2+^ ([Ca^2+^]_e_) entry via voltage-dependent Ca^2+^ channels^25^,^46^. In carp pituitary cells, our recent studies have also demonstrated that NKBa/NKBRPa could stimulate SLα gene expression by NK3R activation via AC/cAMP/PKA, PLC/IP_3_/PKC, and Ca^2+^ /CaM/CaMK-II pathways^14^. Consistent with these previous findings, blocking the respective post-receptor signaling pathways using the AC inhibitor MDL12330A, PKA inactivator H89, PLC blocker U73122, PKC inhibitor GF109203X, removal of [Ca^2+^]_e_ with a Ca^2+^ -free culture medium, and inactivating voltage-sensitive Ca^2+^ channel using nifedipine were all effective in inhibiting/blocking the highly potent synergistic effect on SLα gene expression caused by simultaneous stimulation with SLα, IGF-I and NKBRPa. These results, as a whole, provide evidence that SLα and SLβ can synergize with IGF-I/-II potentiation on SLα gene expression induced by TAC3 gene products by up-regulation of NK3R expression in the carp pituitary.

In summary, we have demonstrated for the first time that SLα and SLβ could act at the pituitary cell level to potentiate the stimulatory effects of TAC3 gene products and IGF-I/-II on SLα gene expression via up-regulation of pituitary NK3R expression. The stimulation on NK3R gene expression probably was mediated through the JAK_2_/STAT_5_, MAPK and PI_3_K/Akt cascades. In this study, we also have a novel findings that simultaneous treatment with SL, IGF and TAC3 gene product together could serve as a highly potent stimulatory signal for SLα gene expression and this stimulatory effect was dependent on NK3R expression in the carp pituitary and involved the activation of post-receptor signaling cascades, namely the AC/cAMP/PKA, PLC/PKC and Ca^2+^ -dependent pathways, coupled to NK3R stimulation. Since SL autoregulation via local release of SLα and SLβ has been recently demonstrated in the carp pituitary^7^. Our findings suggest that SLα and SLβ released at the pituitary level may act in an autocrine/paracrine manner to modulate the pituitary sensitivity to the synergistic stimulation on SLα expression triggered by IGF-I/-II and TAC3 gene products via up-regulation of NK3R expression in the carp pituitary.

## Materials and Methods

### Animals

One-year-old (1^+^) grass carps (*Ctenopharyngodon idellus*) with body weight ranging from 2.0 to 3.0 kg were bought from local markets and maintained in well aerated 250L aquaria at 20 ± 2 °C under a 12L:12D photoperiod. Given the carps at this stage are pre-pubertal and sexual dimorphism is not apparent, fish of mixed sexes were used for preparation of pituitary cell cultures. All animal experiments were conducted in accordance with the guidelines and approval of the respective Animal Research and Ethics committees of the University of Hong Kong and Huazhong Agricultural University.

### Reagents

Recombinant proteins of grass carp SLα and SLβ were expressed in *E. coli*, purified and functionally characterized as described previously^7^. The two hormones were dissolved in PBS and stored frozen at −80 °C as 0.1 mM stocks in small aliquots. Grass carp NKBa and NKBRPa were synthesized from GenScript using the automated solid-phase method, and the carboxyl-terminus of individual peptides was amidated. These peptides were dissoved in DMSO, and stored frozen at −80 °C as 1 mM stocks in small aliquots. Human IGF-I and IGF-II were purchased from Sigma and dissolved in double-distilled deionized water and stored as 0.1 mM stocks in small aliquots at −80 °C. Pharmacological agents, including MDL12330A, H89, GF109203X, U73122, nifedifine, Ly294002, wortmannin, rapamycin, HIMOC, U0126, PD168916, HEX, and IQDMA were acquired from Calbiochem, while senktide and SB222200 were purchased from Tocris. Similar to the peptides, these pharmacological agents were prepared as a high concentration frozen stock in small aliquots and diluted with pre-warmed culture medium to appropriate concentrations 15 min prior to drug treatment.

### Primary culture of grass carp pituitary cells

Grass carp pituitary cells were prepared by trypsin/DNase digestion method as described previously^47^. Briefly, pituitaries were excised from grass carp and diced into 0.5-mm fragments using a McILwain tissue chopper. After 30-min trypsin digestion with constant shaking at 28 °C, pituitary fragments were suspended in Ca^2+^ -free MEM supplemented with DNase I (0.01 mg/ml, Sigma). Pituitary cells were dispersed by gently trituration and filtered through a sterile nylon mesh (pore size: 20 μM) to remove undigested fragments/debris. After that, the cells were harvested by centrifugation at 1000 rpm for 10 min and re-suspended in MEM medium. Total cell yield and percentage viability were estimated by cell counting in the presence of trypan blue using a hematocytometer.

### Measurement of carp SLα and NK3R mRNA expression

Grass carp pituitary cells were seeded in poly-D-lysine coated 24-well culture plates at a density of ~2.5 × 10^6^ /ml/well. On the following day, drug treatment was initiated by replacing the old medium with MEM containing appropriate levels of test substances. After drug treatment, total RNA was extracted from individual well using Trizol and reversely transcribed by Superscript II (50 Unit, Invitrogen). The RT samples obtained were subjected to qPCR using a LightCycler SYBR Green I Kit (Roche) with primers specific for grass carp SLα (Forward Primer: 5′-ACCCACT GTACTTCAATCTCC-3′; Reverse Primer: 5′-CGTCGTAACGATCAAGAGTAG-3′) and NK3R (Forward Primer: 5′-GCCAAGAGAAAGGTTGTGAAGA-3′; Reverse Primer: 5′-GTGTACATGCTGCTCTGGCG-3′), respectively. PCR cycling parameters for SLα and NK3R mRNA detection were set at 94 °C for 3 min followed by 35 cycles of amplification with denaturation at 94 °C for 30 sec, annealing at 52 °C for SLα mRNA or 56 °C for NK3R mRNA for 30 sec, and extension at 72 °C for 30 sec. Signal detection was routinely set for 20 seconds at 84 °C for SLα and 86 °C for NK3R, respectively. In these studies, serial dilutions of plasmid DNA containing the ORF of SLα (GeneBank no: EF372074) and NK3R (GenBank no: JQ254913) cDNA were used as the standards for data calibration. Parallel qPCR measurement of β-actin was also conducted in individual experiment to serve as the internal control.

### Data transformation and statistical analysis

For real-time PCR measurement of NK3R and SLα mRNA, standard curves with a dynamic range of ≥10^5^ and a correlation coefficient ≥0.95 were used for data calibration with RotorGene-Q software 1.7 (Qiagen) under unsupervised mode. Since no significant changes were noted for β-actin mRNA levels between different experiment groups in our studies, SLα and NK3R mRNA data were simply transformed as a percentage of the mean value in the control group without drug treatment (as “%Ctrl”). The data presented (as Mean ± SEM) were pooled results from 6–8 separate experiments and analyzed with ANOVA followed by Dunnett’s test using Prism 6.0 and differences between groups were considered as significant at P < 0.05.

## Additional Information

**How to cite this article**: Hu, G. *et al.* Novel Functional Role of NK3R Expression in the Potentiating Effects on Somatolactin α Autoregulation in grass carp pituitary cells. *Sci. Rep.* **6**, 36102; doi: 10.1038/srep36102 (2016).

**Publisher’s note:** Springer Nature remains neutral with regard to jurisdictional claims in published maps and institutional affiliations.

## Supplementary Material

Supplementary Information
