# Supplementary material for: Novel Functional Role of NK3R Expression in the Potentiating Effects on Somatolactin α Autoregulation in grass carp pituitary cells
Source: Sci Rep. 2016 Oct 27;6:36102. doi: 10.1038/srep36102 (PMC5081563; doi:10.1038/srep36102)
Supplement: Supplementary Information [file srep36102-s1.pdf]

Novel Functional Role of NK3R Expression in the Potentiating Effects on Somatolactin α Autoregulation in grass carp pituitary cells

Guangfu Hu[1, 2]*, Mulan He[2], Anderson On Lam Wong[2]*

1 College of Fisheries, Key Laboratory of Freshwater Animal Breeding, Freshwater Aquaculture Collaborative Innovation Center of Hubei Province, Huazhong Agricultural University, Wuhan, 430070, China

2 School of Biological Sciences, University of Hong Kong, Hong Kong, China

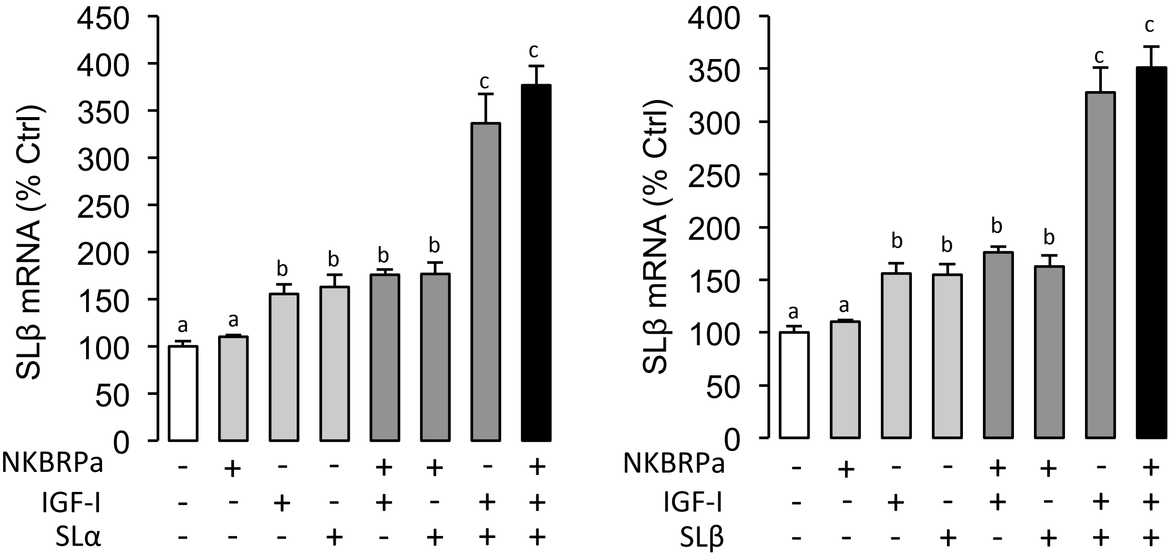

Supplementary Figure. 1 Functional interaction of SLα/β, IGF-I and NKBRPa/senktide on the regulation of SLβ mRNA expression. In this experiment, carp pituitary cells were incubated for 24hr with NKBRPa (1μM), SLα/β (30 nM), IGF-I (50 nM), NKBRPa (1μM)+ SLα/β (30 nM), NKBRPa (1μM) +IGF-I (50 nM), SLα/β (30 nM)+IGF-I (50 nM) or NKBRPa (1μM)+SLα/β (30 nM)+IGF-I (50 nM), respectively. After drug treatment, total RNA was isolated for real-time PCR of SLβ mRNA. In the data present (mean ± SEM), the groups denoted by different letters represent a significant difference at $p < 0.05$ (ANOVA followed by Dunnett's test).
